# Meteorological Register for February, 1817

**Published:** 1817-04

**Authors:** John Bailey

**Affiliations:** Surgeon, &c.


### Meteorological Register for February, 1817,

*Kept at Harwich, in Essex,*

By JOHN BAILEY, Surgeon, &c.

| Moon's Age. | | Atmospherical Preſſure. Morn. | | Ev. | | Temp. | Wind. | Remarks. | General Statement of Diſeaſes. |
|---|---|---|---|---|---|---|---|---|---|
| | 1 | 31 | 3½ | 31 | 3½ | 40 | N | Fine | |
| ○ | 2 | | | | 3 | 39 | N | ditto | |
| | 3 | | 2 | | 1 | 38 | N | Fog | |
| | 4 | 29 | 9 | 29 | 5 | 39 | N | Cloudy | |
| | 5 | | 5½ | | 8 | 40 | SW | Gale of wind | |
| | 6 | | 8 | | 9 | 45 | W | ditto | |
| | 7 | | 3½ | 30 | ½ | 50 | W | ditto | |
| ☾ | 8 | | 2 | | 2 | 48 | W | ditto | |
| | 9 | | 0 | 29 | 9½ | 46 | NW | Fine & clear | |
| | 10 | 29 | 9½ | | | 48 | NW | ditto | |
| | 11 | | 9 | | 8½ | 40 | NE | Rain | |
| | 12 | | 3½ | | 7 | 40 | NW | Snow. Gale of wind | |
| | 13 | | 7½ | | 5½ | 42 | W | Rain | |
| | 14 | | 4 | | 7 | 40 | N | Rain. Gale of wind | |
| | 15 | | 4½ | | 4 | 47 | NW | Rain, ditto | |
| ● | 16 | | 5½ | | 8 | 40 | N | ditto | |
| | 17 | | 8 | | 8 | 52 | NW | | |
| | 18 | 30 | | 29 | 9 | 53 | W | Cloudy | |
| | 19 | | 1 | 30 | 1 | 46 | NW | Very fine | |
| | 20 | 29 | 2½ | 29 | 3½ | 45 | W | Rain. Cloudy | |
| | 21 | | 4½ | | 4½ | 45 | NW | Gale of wind | |
| | 22 | | 5 | | 8 | 41 | NE | ditto | |
| | 23 | | 8½ | | 5 | 40 | W | ditto | |
| ☽ | 24 | | 6 | | 8 | 43 | N | Windy | |
| | 25 | | 9 | | 7 | 50 | W | | |
| | 26 | | 6 | | 7 | 44 | NW | Gale of wind | |
| | 27 | | 4 | | 7 | 44 | NW | Violent gale | |
| | 28 | | 7 | | 7 | 52 | NW | Windy | |

The little rain which has fallen to the earth during the present month, and the prevalence of strong drying winds, have conduced to the healthiness of the season. To this circumstance are to be attributed the freedom from intermittents, and the absence of the more violent forms of pulmonic disorder. A mild kind of ophthalmia, confined chiefly to children, and evidently of an infectious nature, has been frequent, but not to the extent of its last visitation.

